# More Physical Activity, More Work Engagement? A Northern Finland Birth Cohort 1966 Study

**DOI:** 10.1097/JOM.0000000000002530

**Published:** 2022-03-08

**Authors:** Heli Kiema-Junes, Aino Saarinen, Raija Korpelainen, Maarit Kangas, Leena Ala-Mursula, Riitta Pyky, Mirka Hintsanen

**Affiliations:** From the Unit of Psychology, Faculty of Education, University of Oulu, Oulu, Finland (Dr Kiema-Junes and Dr Hintsanen); Department of Psychology and Logopedics, Faculty of Medicine, University of Helsinki, Helsinki, Finland (Dr Saarinen); Department of Sports and Exercise Medicine, Oulu Deaconess Institute Foundation sr, Oulu, Finland; Institute of Health Sciences, University of Oulu and University of Hospital of Oulu, Oulu, Finland (Dr Korpelainen and Dr Pyky); Northern Finland Birth Cohorts, Arctic Biobank, Infrastructure for Population Studies, Faculty of Medicine, University of Oulu, Oulu, Finland (Dr Kangas); Center for Life Course Health Research, University of Oulu, Oulu, Finland (Dr Ala-Mursula).

**Keywords:** accelerometer-measured, leisure time, physical activity, sedentary behavior, sitting time, work engagement

## Abstract

To examine the role of physical activity (PA) and sedentary behavior (SED) for work engagement.

**Methods:**

We used data from Northern Finland Birth Cohort 1966 Study (*n* = 3046 to 4356) to analyze self-reported weekly leisure-time physical activity (LTPA), daily leisure-time sitting time (LTST) and work engagement. PA and SED 24-hour were also measured with accelerometer for 14 days. The data were analyzed using linear regression analyses.

**Results:**

High self-reported LTPA and sports participation were associated with higher work engagement and its subdimensions. High self-reported ST was associated with lower work engagement, vigor, and absorption. Accelerometer-measured light PA was associated with higher work engagement and vigor, and accelerometermeasured steps were linked to higher vigor. Accelerometer-measured SED was associated with lower work engagement, vigor, and dedication.

**Conclusions:**

Self-reported and accelerometer-measured PA and SED may play a role in people’s work engagement.

## Abstract 2

Promoting employees’ leisure-time and total physical activity may promote work engagement although this needs to be confirmed in longitudinal studies. Even light physical activity may foster work engagement, possibly by promoting general psychological well-being and recovery from work. Both self-reported and accelerometer-based physical activity are related to higher work engagement.

Mental requirements are high in contemporary work life, to the extent that every second employee in Europe reports experiencing work-related stress.^1^ Promoting mental health in the workplace is important for decreasing health-related absences, enhancing job performance^2^ and for employees to be able to enjoy working.^3^ Positive psychology has attracted great interest in recent years also in occupational psychology, leading to the concept of work engagement becoming prevalent.^4,5^ Work engagement is defined as a positive emotional and motivational state at work with three sub-dimensions: vigor (high levels of energy, resilience, and persistence), dedication (strong work involvement, sense of significance), and absorption (concentration and being engrossed in one’s work).^6^

Work engagement has many positive outcomes for both employees and organizations. Specifically, work engagement is linked to better employee well-being, health,^7^ work performance^8^ and organizational commitment^9^ resulting in reduced turnover, sickness absenteeism, and disability pensions^10,11^ and organizational productivity.^12,13^ Since work engagement plays a crucial role in working life, research is needed to determine the resources that can boost work engagement. According to Job Demands and Resources (JD-R)—model, the positive physical, psychological, social, and organizational aspects of jobs must be considered to promote work engagement.5 In addition to work and organizational characteristics, positive extra-work factors, such as leisure-time physical activity (LTPA), may be relevant for work engagement.^14^ The concept of LTPA includes both intentional physical exercise and normal daily activities such as playing games or carrying out household chores that require energy expenditure.^15^ So far, the potential effect of higher LTPA on work engagement has received little attention, although it is known to be a major determinant of perceived health.^16–18^ Notably, studies on such associations should consider PA at work which may not imply health gains in a similar way.^19^

Up-to-date, previous evidence has been inconclusive regarding the relationship of PA with work engagement and has mostly concentrated on the linkage between LTPA and mental health, not particularly work engagement although there are few studies suggesting that workplace exercise^20^ and regular exercise^21^ relates to higher work engagement. Also, increased accelerometer-measured sedentary behavior (SED), which refers to any time that a person spends sitting or lying down,^22^ has been shown to be associated with lower work engagement.^23^ On the other hand, many studies have found no evidence that office-based lifestyle interventions can enhance work engagement.^24–26^ Taken together, evidence of an association between LTPA, LTST, total daily PA, accelerometer-measured SED, and work engagement is very limited and contradictory, leading to an apparent need for further studies. Examining associations of PA and engagement have practical significance. In case PA is linked to work engagement, it may provide a means to promote work engagement.

PA has been shown to be linked to mental health through different simultaneously acting mechanisms such as neurobiological, psychosocial, or behavioral^27–28^ that might also underlie an association between PA and work engagement. The hypothesis of neurobiological mechanism suggests that PA promotes psychological health by promoting brain functioning.^29^ A randomized controlled trial study reports of the benefits of exercise training for the growth of hippocampus and memory improvement.^30^ Physical fitness is linked to several aspect of cognitive functioning such as attention, concentration, cognitive flexibility, and processing speed.^31^ Several studies show that physical activity is linked to better cognitive performance,^32,33^ which may act as a potential mechanism between PA and work engagement, especially the dimension of absorption, since it reflects cognitive functioning such as concentration.

Regarding potential psychosocial mechanisms of PA benefits for mental health, the self-determination theory approach suggests that physical activity offers an opportunity to satisfy psychological needs.^34^ PA promotes sense of autonomy by improving self-perception (body image and independence), relatedness by increasing sense of community in sports, and competence by providing mastery and self-efficacy in physical domain as self-efficacy.^27,35^ Additionally, LTPA fosters psychological detachment from work and job recovery that prevents job stress responses and mental disorders in occupational settings^36–38^ and may result in an experience of wellbeing at work^39^ and even work engagement.^40^ PA boosts selfesteem,^41^ optimism^42^ and positive emotions^43^ that are shown to be essential personal resources related to work engagement.^44^

Finally, behavioral mechanism proposes that the benefits of PA for mental health is resulting by changes in relevant and associated behavior such as improvement in sleep duration and sleep efficiency.^27^ Additionally, PA may promote coping and self-regulation skills.^28^

Motivated with these hypotheses of potential underlying mechanisms, the role of PA for work engagement should first be examined on its own. To capture the frequency, intensity, and duration of activity without subjective effect, the accelerometerbased measurement of PA is needed^45^ to complement survey-based data. Moreover, a variety of potential confounding factors, such as gender, marital status, sociodemographic variables, body mass index,^46–48^ work hours,^49^ and stressful work characteristics.^21^

This study aims to examine the relationships of self-reported LTPA and LTST, accelerometer-measured total daily PA and SED with work engagement, taking into account a wide variety of potential confounders. We hypothesized that LTPA and PA are associated with increased work engagement. We also hypothesized that a higher amount of LTST and accelerometer-measured SED is associated with decreased work engagement.

## METHODS

### Participants

This study analyzed both questionnaire-based and clinical data from the 46-year follow-up study of the Northern Finland Birth Cohort (NFBC) 1966,^50,51^ which originally included 12,058 children (with expected birth dates in 1966, representing 96% of all births in the two northernmost provinces in Finland in 1966). In the 46-year follow-up study (target population including those of the original cohort alive and still living in Finland; *n* = 10,321), the NFBC 1966 participants received postal questionnaires. Altogether, 6825 (66%) people answered the questionnaires in 2012 and 5861 (57%) participated clinical examinations in 2012 to 2014. All participants gave their written informed consent before participating in the study. Analyses were run only for those who were currently working full time and only for participants without missing variables, and the final analyses included 3046 to 4356 participants (52.9% male and 47.1% female). Furthermore, the study was approved by the Ethical Committee of the Northern Ostrobothnia Hospital District in Oulu, Finland (94/2011), and the study abided by the Declaration of Helsinki.

### Measures

#### Self-reported LTPA and ST During Leisure Time

LTPA was self-reported in response to questions about the frequency and duration of light PA (described as causing no sweating or breathlessness) and brisk PA (described as causing at least some sweating or breathlessness). The frequency of PA (“How often, and for how long, do you participate in light or brisk physical activity or exercise during your leisure time?”) was reported on a 6-point scale: 1) once a month or less often, 2) two to three times a month, 3) once a week, 4) two to three times a week, 5) four to six times a week, and 6) daily. The duration of PA was also reported on a 6-point scale: 1) not at all, 2) less than 20 minutes, 3) 20 to 39 minutes, 4) 40 to 59 minutes, 5) 1 to 1.5 hours, and 6) more than 1.5 hours.^52^ Weekly averages of light and brisk PA volumes were calculated by multiplying the duration and frequency of both light and brisk levels separately, and total PA was calculated by summing the values for light and brisk PA. The metabolic equivalent of a task (MET) minutes of light and brisk PA were computed by multiplying the PA volume (duration * frequency) by its intensity (3 METs for light PA and 5 METs for brisk PA).

Sports participation was investigated by asking the following question: “How often do you exercise using forms of PA such as walking, cycling, cross-country skiing, swimming, running, strength training, downhill skiing, aerobics, dancing, gymnastics, ball games, motor sports, hiking, picking berries, and hunting?” We also asked about household work such as gardening. All the questionnaire items were answered on a 6-point Likert scale: 1 (not at all), 2 (once a month or rarely), 3 (two to three times a month), 4 (once a week), 5 (two to three times a week), and 6 (four times or more in a week). A mean score was computed for the items.

Participants were asked about their *daily LTST* using the following question: “How much time do you spend sitting on a normal weekday?” ST was evaluated separately according to several domains: “at home,” “watching TV or a video,” “at home in front of computer,” “in a vehicle,” and “in other place.”^53^ We calculated an average score (hours/day) for the items based on accelerometer-measured SED.

#### Accelerometer-Measured PA and SED 24-Hour

Participants attending the clinical examinations for the 46-year follow-up study were asked to use waterproof wrist-worn Polar Active accelerometers to measure their PA.^54^ They were asked to wear the monitor on the non-dominant wrist for 14 days (24 hours per day, including while sleeping) starting after the examination day. Monitors were blinded so as not to give any feedback to the users. Participants were asked to return the monitors by mail after the measurement period. The days with at least 600 minutes of monitoring time were considered valid days for analysis, and participants with four or more valid days were included in the final analyses. The time spent at different activity levels was calculated: *very light physical activity* (VLPA; ie, *sedentary behavior, SED*) corresponded to MET values of 1 to 1.99, light physical activity (LPA; eg, walking or household chores) to MET values of 2 to 3.49, *moderate physical* activity (MPA; eg, brisk walking or light ball games) to MET values of 3.5 to 4.99, *vigorous physical activity* (VPA; eg, jogging) to MET values of 5 to 7.99, and *very vigorous physical activity* (VVPA; eg, running 10 km/h) to MET values of more than 8, respectively. The accelerometers also provided information about *daily steps*, which referred to the mean steps per day of each participant.

#### Work Engagement

The Utrecht Work Engagement Scale (UWES-9)^6^ was used to measure work engagement. The scale consists of three subdimensions (vigor, dedication, and absorption) including nine items (α = 0.9). The subscale for vigor (Cronbach’s α = 0.9) included three items (eg, “In my job, I feel strong and vigorous”); for dedication (α = 0.9), three items (eg, “I am enthusiastic about my job”); and for absorption (α = 0.9), three items (eg, “I feel happy when I am working intensely”). The items were rated on a 6-point Likert scale ranging from 0 (never) to 6 (always). Work engagement was calculated as the average of the items. The validity and reliability of the scale were confirmed by a previous study, and the scale has been widely used for measuring work engagement.^55^

### Covariates

Gender was dichotomized as male or female. Marital status was coded as 1) not in a relationship (single, divorced, or widowed) or 2) in a relationship (married, cohabiting, or in a registered partnership). Education level was coded as follows: 1) comprehensive school, 2) intermediate, and 3) university-level education. Socioeconomic status was classified as 1) manual, 2) low-level non-manual, and 3) high-level non-manual.

*Body mass index* (BMI, kg/m^2^) was used as a control variable since it is associated with PA^46^ and work engagement.^47^ BMI was computed by dividing each participant’s body weight by his/her squared body height.^48^

*Work hours* were assessed by asking the participants, “How many hours approximately do you work in a week?” The question was answered on a 7-point scale: 1) 1 to 10 hours, 2) 11 to 20 hours, 3) 21 to 30 hours, 4) 31 to 40 hours, 5) 41 to 50 hours, 6) 51 to 60 hours, and 7) over 60 hours. The analyses were adjusted for the number of work hours, which may have acted as a confounder.^49^

Job strain and effort–reward imbalance were used as indicators of stressful job characteristics. *Job strain* was measured using a modified Job Content Questionnaire (JCQ).^56^ Job strain was computed by dividing the score for the job demand items (mean of 4 items on a 5-point Likert scale; eg, “My job requires readiness for fast action”) by the score for the job control items (mean of 9 items on a 5-point Likert scale; eg, “I can influence my work tasks”). High values for this ratio reflected high job strain, resulting from high demands combined with low job control.^56^

*Effort–reward imbalance* was assessed using the Occupational Stress Questionnaire.^57^ Effort was measured with three items (eg, “I have constant time pressure due to heavy workload,” Cronbach’s α = 0.7) and reward with seven items, three of which were reversed (eg, “I receive the respect I deserve from my superiors,” α = 0.7). Effort items reflected the time and energy employees invested in their jobs, whereas reward items measured career opportunities, including job security, amount of salary, and esteem at work.^57^ The effort–reward imbalance was computed by dividing the mean score for effort by the mean score for reward.

The survey variables were measured in 2012, and the clinical measurements took place in 2012 to 2014, comprising the 46-year follow-up of the 1966 NFBC Study.

### Statistical Analyses

First, we examined whether gender modified the associations between the PA measures and work engagement and its subdimensions. When predicting work engagement, vigor, and dedication, the interaction of gender with accelerometer-measured light intensity PA was significant (for work engagement *P* = 0.006; for vigor *P* = 0.016; for dedication *P* = 0.006). The interaction of gender with accelerometer-measured SED yielded a significant association for vigor (*P* < 0.001). All the other gender interactions were non-significant; thus, we ran the analyses for the total sample, including both men and women, and additionally analyses were run separately for men and women for those associations with significant gender interactions. A table illustrating the gender-stratified analyses for the relationships of accelerometer-measured SED with work engagement and light PA with work engagement can be found in the Supplementary Table 2, http://links.lww.com/JOM/B79.

Second, we conducted linear regression analyses to examine the associations of self-reported LTPA, self-reported LTST, accel-erometer-measured PA, and accelerometer-measured SED with work engagement. Dependent variables included the total score for work engagement and its three subdimensions. We conducted separate analyses for each dependent variable. Independent variables included five self-reported measures (light LTPA volume [min/week], brisk LTPA [min/week], total LTPA [min/week], sports participation, and ST) and six accelerometer-measured PA variables (very light PA/accelerometer-measured SED [min/day], light PA [min/day], moderate PA [min/day], vigorous PA [min/day], very vigorous PA [min/day], and steps). The variables for self-reported LTPA, self-reported LTST, accelerometer-measured PA, and accelerometer-measured SED were analyzed separately as independent variables. In the analyses, we used standardized values for the self-reported and accelerometer-measured PA variables and accelerom-eter-measured SED and LTST (mean = 1, SD = 0).

Model 1 was adjusted for gender, and Model 2 was fully adjusted (ie, adjusted for gender, marital status, socioeconomic status, education, work hours, body-mass index, and stressful job characteristics).

## RESULTS

The descriptive statistics for the study variables are presented in Table 1.

**TABLE 1:** Descriptive Statistics

Variable (Measurement Range)	*N*	Mean	SD	Frequency (%)
Gender (0–1)	4818			
Women				2087 (47.1%)
Marital status (0–1)				
In a relationship				3491 (80.3%)
Educational level (0–2)	4330			
Comprehensive				12 (0.3%)
Intermediate				2221 (51.3%)
Academic level education				2109 (48.7%)
Socioeconomic status (0–3)	4372			
manual				2134 (48.8%)
Lower non-manual				1088 (24.9%)
Upper non-manual				1150 (26.3%)
Body-mass Index (km/m^2^)	4322	26.45	0.04	
Work hours (1–7)	4356	4.25	0.72	
Job strain (1–5)	3475	2.80	1.15	
Effort-Reward Imbalance (1–4)	4350	2.63	1.15	
Work engagement (0–6)	4349	4.63	1.20	
Vigor (0–6)	4349	4.69	1.18	
Dedication (0–6)	4348	4.58	1.33	
Absorption (0–6)	4347	4.37	1.42	
Self-reported LPA				
Light LTPA (min/week)	4208	163.11	160.48	
Brisk LTPA (min/week)	4318	117.97	120.33	
Total LTPA (min/week)	4173	276.79	229.71	
Sport participation (1-6)	3629	1.96	0.38	
Sitting time (hour/day)	4195	3.9	2.13	
Accelerometer-measured PA				
Sedentary behavior	3525	636.82	88.55	
Light PA (min/day)	3525	274.42	68.33	
Moderate PA (min/day)	3525	37.42	21.88	
Vigorous PA (min/day)	3525	23.97	16.03	
Very vigorous PA (min/day)	3525	8.70	10.51	
Steps (mean/day)	3366	10,707.58	3506.32	

SD, standard deviation.

Bivariate associations (Pearson’s correlation coefficients) are presented in Supplementary Table 1, http://links.lww.com/JOM/B80. The subdimensions of work engagement were positively correlated with each other and with the total score for work engagement. High body mass index correlated with lower self-reported LTPA (*r* = –0.12, *P* < *0.001*) and with lower accelerometer-measured PA levels (LPA *r* = –12, *P* < *0.001*; MPA *r* = –0.05, *P* < *0.001*; VPA *r* = –0.18, *P* < *0.001* and VVPA *r* = –0.12, *P* < *0.001*). Accelerometer-measured light PA and moderate PA were positively correlated (*r* = 0.30, *P* < *0.001*). Similarly, accelerometer-measured vigorous and very vigorous PA were positively correlated (*r* = 0.12, *P* < *0.001*) with each other. Self-reported LTPA was positively correlated with accelerometer-measured VVPA (*r* = 0.27, P < *0.001*) and steps (*r* = 0.27, *P* < *0.001*). Self-reported ST at work correlated positively with total self-reported sitting time (*r* = 0.535, *P* < *.001*), but negatively with self-reported sitting time during leisure time (*r* = -0.111, *P* < *.001*).

### Associations Between Self-Reported PA, LTST During Leisure Time and Work Engagement

Table 2 presents the results of the gender-adjusted linear regression analyses for predicting work engagement by self-reported LTPA and LTST. High self-reported light and brisk LTPA were associated with higher total work engagement (light *B* = 0.07, *P* < *0.001*; brisk *B* = 0.11, *P* < *0.001*), vigor (light *B* = 0.09, *P* < *0.001*; brisk *B* = 0.12, *P* < *0.001*), dedication (light *B* = 0.05, *P* = 0.009; brisk *B* = 0.11, *P* < *0.001*), and absorption only for brisk LTPA (*B* = 0.09, *P* < *0.001*). High self-reported total LTPA was also associated with higher total work engagement (*B* = 0.11, *P* < *0.001*), vigor (*B* = 0.13, *P* < *0.001*), dedication (*B* = 0.10, *P* < *0.001*), and absorption (*B* = 0.08, *P* < *0.001*). High self-reported sports participation was associated with higher work engagement (*B* = 0.17, *P* < *0.001*), vigor (*B* = 0.18, *P* < *0.001*), dedication (*B* = 0.16, *P* < *0.001*), and absorption (*B* = 0.13, *P* < *0.001*). Self-reported LTST was slightly associated with lower engagement (*B* = –0.06, *P* = *0.024*), vigor (*B* = –0.07, *P* = *0.003*) and lower absorption (*B* = –0.06, *P* = *0.031*).

**TABLE 2:** Result of Gender-Adjusted and Fully Adjusted Linear Regression Analyses When Examining the Associations of Self-Reported Leisure Time Physical Activity and Sitting Time with Work Engagement

						The Subscales of Work Engagement														
	Work Engagement					Vigor					Dedication					Absorption				
	*B*	*SE*	*Adj.R* ^2^	*R*^2^ Change	*P*	*B*	*SE*	*AdjR* ^2^	*R*^2^ Change	*P*	*B*	*SE*	*AdjR* ^2^	*R*^2^ Change	*P*	*B*	*SE*	*AdjR* ^2^	*R*^2^ Change	*P*
*Model 1*																				
Light LTP*	0.072	0.019	0.019	0.004	<0.001	0.087	0.018	0.019	0.005	<0.001	0.054	0.021	0.014	0.002	0.009	0.040	0.022	0.012	0.001	0.073
Brisk LTP*	0.111	0.018	0.023	0.009	<0.001	0.120	0.018	0.023	0.010	<0.001	0.105	0.020	0.017	0.006	<0.001	0.085	0.022	0.014	0.004	<0.001
Total LTP*	0.114	0.019	0.024	0.009	<0.001	0.127	0.019	0.025	0.011	<0.001	0.100	0.021	0.017	0.006	<0.001	0.080	0.023	0.013	0.003	<0.001
*Sport participation*	0.171	0.020	0.038	0.020	<0.001	0.176	0.020	0.039	0.022	<0.001	0.164	0.022	0.030	0.015	<0.001	0.126	0.024	0.021	0.008	<0.001
*Sitting time*	–0.055	0.024	0.016	0.002	0.024	–0.071	0.024	0.014	0.003	0.003	–0.025	0.027	0.013	0.000	0.347	–0.063	0.029	0.011	0.002	0.031
*Model* 2																				
Light LTP*	0.078	0.020	0.130	0.004	<0.001	0.088	0.020	0.115	0.005	<0.001	0.063	0.022	0.119	0.002	0.005	0.051	0.025	0.080	0.001	0.040
Brisk LTP*	0.086	0.020	0.130	0.005	<0.001	0.104	0.020	0.116	0.007	<0.001	0.076	0.022	0.118	0.003	<0.001	0.053	0.025	0.081	0.001	0.035
Total LTP*	0.107	0.020	0.136	0.008	<0.001	0.123	0.020	0.122	0.010	<0.001	0.094	0.023	0.123	0.005	<0.001	0.071	0.025	0.082	0.002	0.005
*Sport participation*	0.156	0.021	0.141	0.016	<0.001	0.161	0.021	0.126	0.017	<0.001	0.147	0.024	0.071	0.013	<0.001	0.113	0.027	0.084	0.006	<0.001
*Sitting time*	0.003	0.027	0.117	0.000	0.902	–0.027	0.027	0.102	0.000	0.319	0.043	0.030	0.109	0.001	0.147	0.010	0.034	0.067	0.000	0.766

Model 1 was adjusted for gender. Model 2 was fully adjusted (adjusted for gender, marital status, education, socioeconomic status, work hours, body-mass index, effort-reward imbalance, and job strain.

*Leisure time physical activity *n* = 3561–4234.

For Model 2 (Table 2), the associations were adjusted for gender, marital status, socioeconomic status, education, work hours, body mass index, effort–reward imbalance, and job strain. All the associations remained significant, except for the associations between self-reported LTST and work engagement and its subdimensions. Variables for self-reported LTPA explained 1% to 2% of the variation in work engagement.

### Associations of Accelerometer-Measured PA and SED 24-Hour with Work Engagement

Table 3 shows the results for the associations of accelerometer-measured PA and SED with work engagement. In the gender-adjusted analyses, LPA was associated with higher work engagement (*B* = 0.05, *P* = *0.027*) and higher vigor (*B* = 0.08, *P* < *0.001*). Additionally, LPA related to higher work engagement (*B* = 0.094, *P* < *0.001*), vigor (*B* = 0.118, *P* < *0.001*), and dedication (*B* = 0.081, *P* = 0.005) among women, but not among men, in separate analyses. MPA was associated with lower dedication (*B* = –1.20, *P* = *0.028*) and absorption (*B* = –1.19, *P* = *0.042*). Accelerometer-measured steps were also associated with higher vigor (*B* = 0.06, *P* = *0.002*). High accelerometer-measured SED related to lower vigor (*B* = –5.99, *P* < *0.001*), and SED was linked to lower work engagement (*B* = –7.299, *P* = 0.002), vigor (*B* = –9.990, *P* <*0.001*), and dedication (*B* = –5.704, *P* = *0.032*), especially among women, but not among men.

**TABLE 3:** Gender and Fully Adjusted Linear Regression Analyses When Examining the Associations of Accelerometer-Measured Physical Activity and Sedentary Time with Work Engagement

						The Subscales of Work Engagement														
	Work Engagement					Vigor					Dedication					Absorption				
	*B* ^a^	*SE*	*Adj· R* ^2^	*R* ^2^ *change*	*P*	*B*	*SE*	*AdjR* ^2^	*R* ^2^ *change*	*P*	*B*	*SE*	*AdjR* ^2^	*R* ^2^ *change*	*P*	*B*	*SE*	*AdjR* ^2^	*R* ^2^ *change*	*P*
*Model* 1																				
*SED*^b^*(min/day)*	–2.978	1.779	0.017	0.001	0.094	–5.989	0.000	0.019	0.003	<0.001	–1.070	1.797	0.013	0.000	0.589	2.675	2.112	0.013	0.000	0.205
*Light PA*^c^*(min/day)*	0.045	0.020	0.018	0.001	0.027	0.076	0.020	0.020	0.004	<0.001	0.026	0.022	0.013	0.000	0.244	–0.025	0.024	0.013	0.000	0.302
*Moderate PA*^c^*(min/day)*	–0.755	0.494	0.017	0.001	0.126	–0.231	0.488	0.016	0.000	0.635	–1.204	0.549	0.014	0.001	0.028	–1.192	0.586	0.013	0.001	0.042
*Vigorous PA*^c^*(min/day)*	–0.009	0.338	0.016	0.000	0.978	0.390	0.333	0.016	0.000	0.243	–0.489	0.376	0.013	0.000	0.193	–0.276	0.401	0.012	0.000	0.491
*Very Vigorous PA*^c^*(min/day)*	0.198	0.221	0.017	0.000	0.371	0.321	0.218	0.016	0.001	0.141	0.058	0.245	0.013	0.000	0.812	0.055	0.262	0.012	0.000	0.835
*Steps (steps/day)*	0.026	0.021	0.017	0.000	0.208	0.064	0.020	0.019	0.003	0.002	–0.005	0.023	0.012	0.000	0.822	–0.039	0.025	0.012	0.001	0.111
*Model 2*																				
*SED*^b^*(min/day)*	–5.882	1.994	0.128	0.003	0.003	–7.362	1.996	0.113	0.004	<0.001	–4.333	2.216	0.118	0.001	0.051	–2.903	2.475	0.078	0.000	0.241
*Light PA*^c^*(min/day)*	0.061	0.023	0.127	0.002	0.008	0.081	0.023	0.113	0.004	<0.001	0.041	0.025	0.117	0.001	0.107	0.024	0.028	0.077	0.000	0.400
*Moderate PA*^c^*(min/day)*	–0.276	0.520	0.125	0.000	0.595	0.132	0.521	0.109	0.000	0.800	–0.706	0.577	0.117	0.000	0.221	–0.365	0.644	0.077	0.000	0.570
*Vigorous PA*^c^*(min/day)*	0.077	0.360	0.125	0.000	0.831	0.378	0.361	0.109	0.000	0.295	–0.280	0.400	0.117	0.000	0.485	–0.092	0.447	0.077	0.000	0.837
*Very Vigorous PA*^c^*(min/day)*	0.080	0.237	0.125	0.000	0.734	0.199	0.237	0.109	0.000	0.402	–0.080	0.263	0.116	0.000	0.760	–0.015	0.293	0.077	0.000	0.960
*Steps (steps/day)*	0.023	0.023	0.125	0.000	0.307	0.051	0.022	0.110	0.002	0.025	–0.004	0.025	0.115	0.000	0.887	–0.013	0.028	0.075	0.000	0.649

^a^Unstandardized beta. Model 1 was adjusted for gender. Model 2 was fully adjusted (adjusted for gender, marital status, education, socioeconomic status, work hours, body-mass index, effort-reward imbalance, and job strain).

^b^Sedentary behavior.

^c^Physical activity, *n* = 3315–3461.

In the fully adjusted analyses, the results remained almost identical for SED and vigor, light PA and vigor, and steps and vigor. The fully adjusted model also showed a positive association between LPA and dedication (*B* = 0.07, *P* = *0.005*). In separate analyses for men and women, LPA was positively linked to work engagement (*B* = 0.093, *P* < 0.001), vigor (*B* = 0.107, *P* < *0.001*), and dedication (*B* = 0.081, *P* = *0.010*) among women, but with higher vigor among men (*B* = 0.075, *P* = *0.027*). High accelerometer-measured SED was associated with lower engagement (*B* = – 0.001, *P* < *0.001*) and dedication (*B* = –0.001, *P* = *0.024*). In the fully adjusted model, LPA was positively linked to engagement (*B* = 0.061, *P* = *0.008*) and dedication (*B* = 0.081, *P* < *0.001*). Accelerometer-measured steps were also associated with higher vigor (*B* = 0.05, *P* = *0.025*) in the fully adjusted model. Unlike the gender-adjusted model, the fully adjusted model did not yield any significant associations for MPA with dedication and absorption. Accelerometer-measured measures of PA and accelerometer-measured SED did not explain any variation in work engagement when fully adjusted.

As additional analyses, we examined whether PA at work and physical job demands could act as confounders for the association between PA and work engagement; thus, in the additional analyses, we included those factors as covariates. All the significant associations found in Model 1 remained significant.

## DISCUSSION

This large population-based study revealed that self-reported LTPA and sports participation in leisure time were consistently linked to higher work engagement and its subdimensions, whereas self-reported sitting time in leisure time related to lower work engagement and its subdimensions, except for dedication. We found that accelerometer-measured light PA 24-hour related to higher work engagement and vigor, and accelerometer-measured steps 24-hour were associated with vigor. Additionally, accelerometer-measured SED 24-hour was associated with lower work engagement, vigor, and dedication.

Our results for self-reported LTPA and work engagement are new contributions to the literature. Previously, Van Berkel et al^26^ found no significant association between self-reported PA and work engagement. The sample included only 257 employees and was restricted to workers in research institutes, whereas our sample covered 6825 participants from all branches of working life in the private and public sectors, which constitutes a major distinction between these two studies. Another study indicated a positive association between off-job activities (with PA as one option) and feelings of vigor and work engagement the following day at work.^58^ Other factors, such as psychological detachment from work and feelings of relaxation,^24,43,58^ may partly explain the relationship between PA and work engagement, but this needs to be further examined. The role of psychological detachment has also been emphasized by scholars, especially in the relationship between PA and vigor.^43,59^ PA during workdays and after work is associated with successful recovery and detachment from work, which prevents exhaustion and promotes well-being, and especially vigor, at work.^60,61^ Additionally, LTPA promotes a positive recovery experience that buffers the effect of work-and-family conflict on work engagement.^14^ One study reported that employees who exercise regularly tend to have higher levels of vigor during physical exercise, which is linked to high well-being at work.^62^ Furthermore, physical activities offer a way to satisfy psychological demands, such as the need for competence and relatedness.^34,43^

Our study showed robust associations between self-reported LTST and lower work engagement, vigor, and absorption. These results aligned with prior studies on physical inactivity^63^ and accelerometer-measured SED^23,64,65^ and their role in decreasing levels of work engagement. Additionally, our study revealed that LTST was linked to higher absorption when adjusting for gender, but in the fully adjusted model, the association did not remain significant. This was in line with a previous study that reported that both men and women with high work engagement are less likely to have prolonged accelerometer-measured SED, but women with high levels of absorption are more likely to have prolonged accelerometer-measured SED.^64^ Since evidence for the association between accelerometer-measured SED and work engagement is still lacking, with only a few studies covering the subject,^23,65^ the connection needs to be examined in further studies.

Our study showed associations between accelerometer-measured light PA and higher work engagement, vigor, and dedication. Accelerometer-measured steps were also linked to higher vigor, but accelerometer-measured SED was associated with lower work engagement, vigor, and dedication. These results accord with previous research regarding the link between leisure time PA and well-being outcomes.^66,67^ The effect of PA on general well-being,^68^ work engagement,^21^ and vigor^59^ has been covered in the literature, but with few exceptions,^24–26^ evidence for the effect of PA on work engagement is still almost non-existent. Its stress-buffering effect is an important outcome of PA,^36^ and work engagement researchers have highlighted the emotional benefits of PA and its impact on successful recovery from work.^69^ According to one study, subjective experience of PA plays a crucial role in the recovery-from-work process, rather than the actual time spent engaging in physical activities,^69^ which also explains the discrepancy in our study between self-reported brisk LTPA and accelerometer-measured vigorous PA; self-reported brisk PA was linked to higher work engagement and its subdimensions, but accelerometer-measured vigorous PA had no significant association with work engagement or its subdimensions. Moreover, the accelerometers measured all activity 24 hours a day, while LTPA was restricted to covering leisure-time activities.

A review study indicated that light PA is especially important for both physical and psychological health, since it has a pronounced negative association with accelerometer-measured SED.^70^ Accelerometer-measured SED and LTST were both negatively associated with work engagement in our study, which agreed with previous research indicating a link between work-related accelerometer-measured SED and lower work engagement.^23^ SED was linked to lower work engagement, vigor, and dedication, especially among women, and prior studies have indicated that male office workers simply spend proportionally more time sitting than female office workers.^70^

Overall, it seems that light PA offsets accelerometer-measured SED,^71^ which particularly promotes health in general.^72,73^ Accelerometer-measured moderate PA related to lower dedication and absorption in the gender-adjusted model, but in the fully adjusted model, the associations were not significant. A prior study indicated that objectively measured PA is not significantly associated with well-being outcomes or with work engagement.^26^ Our study showed associations between light PA and work engagement, vigor, dedication, accelerometer-measured steps, and vigor, but other activity levels measured with accelerometers had no robust associations; therefore, our study also showed that accelerometer measured steps were linked to higher vigor. A recent study indicated that objectively measured PA may be associated particularly with vigor^20^ but not with other subdimensions or total work engagement, so our study supports this earlier research. Additionally, gender modified the association between work engagement, vigor, dedication, and light PA; light PA was linked to higher work engagement, higher vigor, and higher dedication among women, but not among men. Prior studies have indicated that men are more physically active than women, especially based on vigorous PA,^74^ but women engage in lighter-intensity PA and are more active according to total volume of PA.^75^

The results for self-reported PA and accelerometer-measured PA differed from one another. Analyses of the self-reported PA produced highly uniform and robust findings, whereas the accelerometer-measured analyses indicated links only between light PA and work engagement, between steps and vigor, and between accelerometer-measured SED and work engagement. Also, previously objectively measured PA has shown fewer associations with psychological health Hamer and Stamatakis^76^ reported no significant association between objectively measured PA and psychological health, despite a strong link between self-reported moderate PA and psychological well-being. One main explanatory factor for this difference is that all the self-reported surveys examined only PA in leisure time, whereas the accelerometers in this study measured different activity levels from very light to very vigorous PA over 24 hours, thus also including work-time activity. Self-reports indicate an individual’s own impressions and perceptions of activity levels, whereas accelerometers and objective methods can precisely measure amounts of activity.^77^ Self-report measures of moderate and vigorous PA are closely related to a person’s fitness, whereas accelerometers measure moderate and vigorous activity independently of fitness.^78^ Self-reports tend to overestimate the amount of PA and underestimate the amount of ST.^78^

Additionally, the amount and level of physical activity differs in terms of leisure time versus working time. PA in leisure time was consistently linked to higher work engagement and its subdimensions, whereas only accelerometer-measured 24-hour light PA and steps showed positive association with work engagement and vigor. Self-reported sitting time at work correlated with total self-reported sitting time, but negatively with self-reported sitting time during leisure time. It may be, that work, which requires long sitting hours produces increased need for physical activity during leisure time, especially in terms of psychological detachment and recovery process.^69^ Previous population-based studies have shown an inverse association between PA during leisure time and morbidity and even mortality especially among older adults.^79–81^

Self-reported ST was associated with lower work engagement, vigor, and absorption in the gender-adjusted model, although these association were not apparent in the fully adjusted analysis. Overall, our study indicated that accelerometer-measured SED and ST are linked with lower work engagement, and our results aligned with prior studies reporting a negative effect of accelerometer-measured SED on well-being and work engagement.^23^

Overall, the effect sizes remained quite low; however, it is expected that accelerometer-measured PA and SED can explain only a small part of the variance in work engagement, since many other factors, based on the plausible multiple mechanisms underlying the association, can influence work engagement.^83^

### Limitations, Strengths, Future Directions, and Practical Implications

The most important limitation of this study is its cross-sectional design, making it impossible to draw causal inferences between PA, accelerometer-measured SED, and work engagement. Another limitation is its predominant use of self-report measures, which pose a risk of common method bias.^83^ However, the linkage to objective measures of PA supported the results obtained with self-reports. Another limitation of this study is, that the measures of this study indicated PA in different contexts (leisure time, 24-hour) and thus, decrease comparability between the measures.

The strengths of the study relate to the large non-selective population-based birth cohort sample covering all branches of working life in both the public and private sectors. The combination of self-reported and accelerometer measured PA is an important strength of this study and improves its validity. The well-validated measures of work engagement,^54^ leisure-time PA,^52^ and ST^84^ that we used in this study strengthened the reliability of the results, and we also adjusted for many potentially confounding factors.

Future studies should examine the association between PA and work engagement longitudinally. The association between PA and work engagement is probably quite complex, since several mediating and moderating factors such as psychological detachment or sense of belonging are involved^24,43,40,58,60,61^ in the link between PA and work engagement, which should be examined further.

## CONCLUSIONS

To summarize, this study provides insights into the role of overall PA and SED in the experience of work engagement. The study highlights the importance of the linkage between PA, sports participation, and work engagement. Additionally, it implies a need to reduce ST in order to improve work engagement. Our findings suggest that promoting PA and reducing ST are vital for work engagement. Even light PA may be beneficial for work engagement, since it is closely related to lesser amounts of ST;^70^ however, our results should be confirmed by a longitudinal study to identify causal associations between PA, accelerometer-measured SED, and work engagement.
